# Epigenetic and transcriptional regulation of CCL17 production by glucocorticoids in arthritis

**DOI:** 10.1016/j.isci.2023.108079

**Published:** 2023-09-27

**Authors:** Tanya J. Lupancu, Kevin M.C. Lee, Mahtab Eivazitork, Cecil Hor, Andrew J. Fleetwood, Andrew D. Cook, Moshe Olshansky, Stephen J. Turner, Richard de Steiger, Keith Lim, John A. Hamilton, Adrian A. Achuthan

**Affiliations:** 1 Department of Medicine, Royal Melbourne Hospital, The University of Melbourne, Parkville, VIC 3052, Australia; 2 Department of Medicine, Western Health, The University of Melbourne, St Albans, VIC 3021, Australia; 3 Haematopoiesis and Leukocyte Biology, Baker IDI Heart and Diabetes Institute, Melbourne, VIC 3004, Australia; 4 Department of Microbiology, Monash University, Clayton, VIC 3800, Australia; 5 Department of Surgery, Epworth HealthCare, The University of Melbourne, Richmond, VIC 3121, Australia

**Keywords:** Orthopedics, Molecular mechanism of gene regulation, Epigenetics

## Abstract

Glucocorticoids (GCs) are potent anti-inflammatory agents and are broadly used in treating rheumatoid arthritis (RA) patients, albeit with adverse side effects associated with long-term usage. The negative consequences of GC therapy provide an impetus for research into gaining insights into the molecular mechanisms of GC action. We have previously reported that granulocyte-macrophage colony-stimulating factor (GM-CSF)-induced CCL17 has a non-redundant role in inflammatory arthritis. Here, we provide molecular evidence that GCs can suppress GM-CSF-mediated upregulation of IRF4 and CCL17 expression via downregulating JMJD3 expression and activity. In mouse models of inflammatory arthritis, GC treatment inhibited CCL17 expression and ameliorated arthritic pain-like behavior and disease. Significantly, GC treatment of RA patient peripheral blood mononuclear cells *ex vivo* resulted in decreased CCL17 production. This delineated pathway potentially provides new therapeutic options for the treatment of many inflammatory conditions, where GCs are used as an anti-inflammatory drug but without the associated adverse side effects.

## Highlights

• Dexamethasone suppresses GM-CSF-induced CCL17 formation in monocytes and macrophages
• By suppressing JMJD3/IRF4 expression, dexamethasone inhibits CCL17 expression
• Decreasing CCL17 correlates with dexamethasone-mediated amelioration of arthritis
• Dexamethasone-mediated inhibition of CCL17, IRF4, and JMJD3 is evident in RA samples

## Graphical abstract

**Figure:**
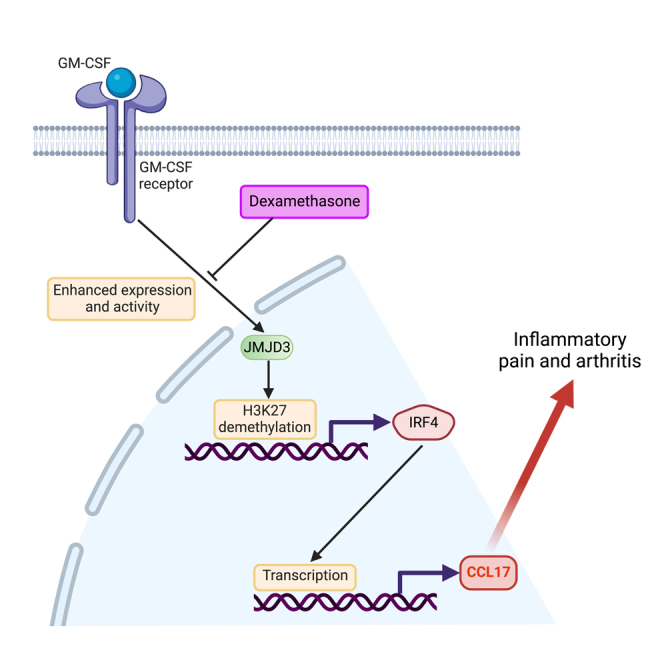

## Introduction

Rheumatoid arthritis (RA) is a chronic inflammatory autoimmune disease, which leads to poor quality of life due to the debilitating effect of inflammation which, if left untreated, causes irreversible joint damage. There is no known cure for RA, and current treatments aimed at managing the disease are costly and accompanied by significant side effects, as well as limited effectiveness in some patients,^1^ highlighting the urgent need for new therapeutics.

Glucocorticoids (GCs) are potent anti-inflammatory and immunosuppressive agents broadly used as first-line anti-inflammatory therapy, despite their well-known side effects linked to their prolonged usage. Recent American College of Rheumatology (ACR) and European League Against Rheumatism (EULAR) recommendations on RA management advocate the use of GCs as adjunct treatment to conventional disease-modifying antirheumatic drugs (cDMARDs) at the lowest concentration and the shortest time possible.^2^ GC-mediated inhibition of inflammation is believed to occur through transcriptional activation of anti-inflammatory proteins (e.g., interleukin 10 [IL10]), as well as through repression of proinflammatory transcription factors (e.g., nuclear factor κB [NF-κB]). Unfortunately, such broad transcriptional activities of GCs can lead to an increased risk of infections and osteoporosis.^3^

Clinical trials in RA targeting the cytokine, granulocyte-macrophage colony-stimulating factor (GM-CSF), are showing promise although its mode of action remains largely unknown.^4^^,^^5^ We have previously reported that GM-CSF drives CCL17 production via an interferon regulatory factor 4 (IRF4)-dependent pathway in human monocytes and mouse macrophages.^6^ Utilizing several inflammatory models of arthritis, we have demonstrated that CCL17 has a non-redundant role in mediating arthritic pain-like behavior and disease.^6^^,^^7^^,^^8^^,^^9^ While the GC-mediated negative regulation of inflammatory cytokines, such as tumor necrosis factor (TNF), has been widely studied, the molecular regulation of CCL17 production by GCs has not yet been investigated.

In the present study, we identified a novel molecular mechanism of anti-inflammatory action by GCs in human monocytes and mouse macrophages. Significantly, we report here that GC treatment can suppress GM-CSF-induced CCL17 production in peripheral blood mononuclear cells (PBMCs) from RA patients as well as in synovial cells from an inflammatory arthritis mouse model. We provide molecular evidence for the first time that GCs can inhibit the GM-CSF-upregulated IRF4 transcription factor, which is required for CCL17 production, via the downregulation of the expression and activity of Jumonji D3 (JMJD3 or gene name *KDM6B*) demethylase. GC-mediated suppression of JMJD3 function results in suppression of IRF4 transcription due to the presence of the repressive histone 3 lysine 27 trimethylation (H3K27me3) epigenetic modification in the IRF4 gene transcription start site (TSS). Given the adverse side effects associated with long-term usage of GCs, we propose that selective targeting of downstream mediators, such as CCL17, would be beneficial in treating chronic inflammatory conditions.

## Results

### Dexamethasone inhibits GM-CSF-induced CCL17 production in human monocytes and mouse macrophages

**Figure 1:**
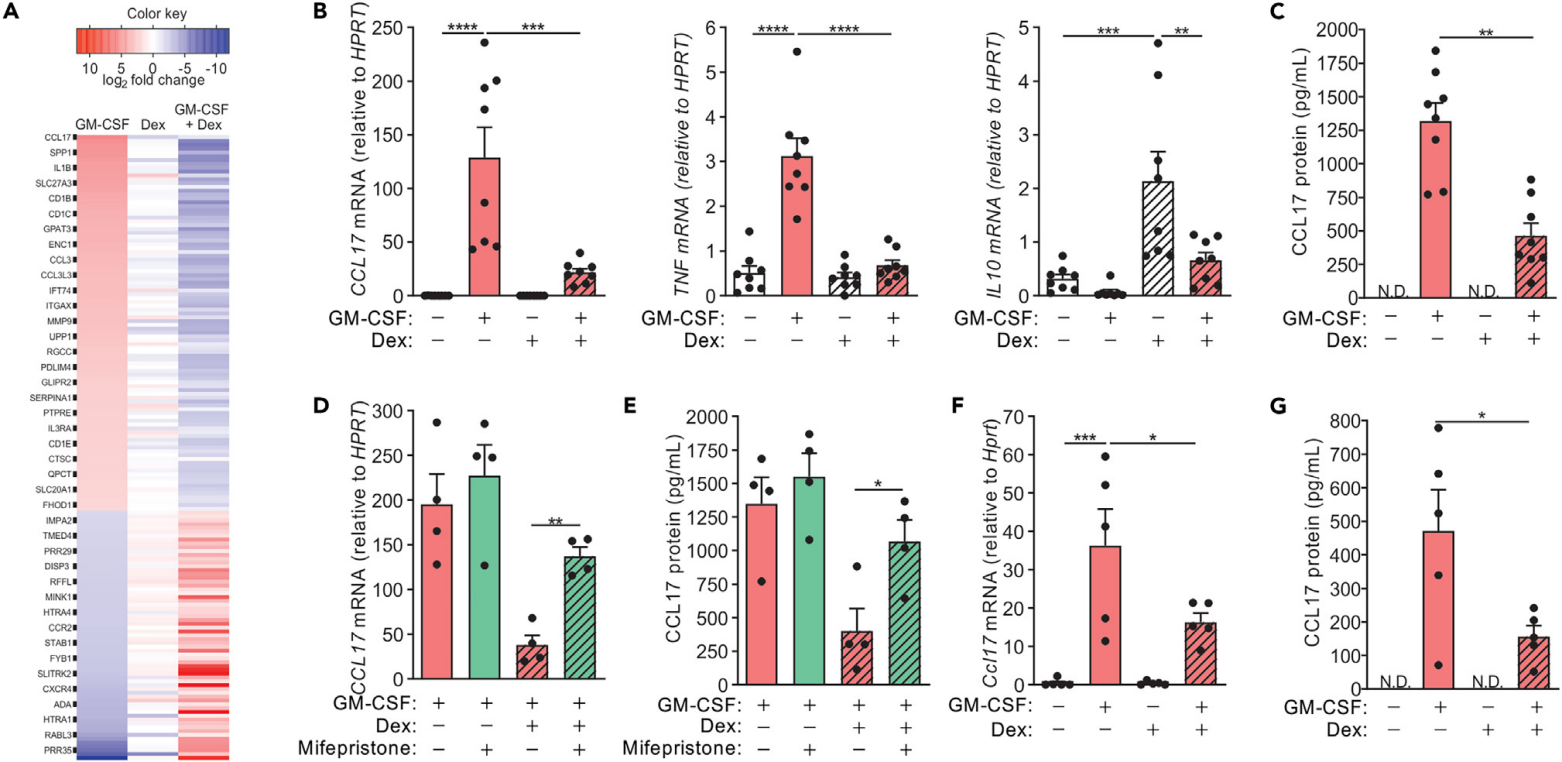
Dexamethasone inhibits GM-CSF-induced CCL17 production in human monocytes and mouse macrophages (A–C) Human monocytes were treated with either GM-CSF (20 ng/mL) and Dex (100 nM) alone or together for 16 h. A) Heatmap of significantly (FDR<0.05) regulated genes (n = 3; for simplicity, every 4^th^ protein coding gene name is labeled in the heatmap and the comprehensive list of genes is provided in Table S1). B) mRNA expression (qPCR) and C) secreted CCL17 protein (ELISA) (n = 8). (D and E) Human monocytes were pre-treated with mifepristone (1 μM) for 30 min before treated with GM-CSF (20 ng/mL) alone or together with Dex (100 nM) for 16 h. D) *CCL17* mRNA expression and E) secreted CCL17 protein (n = 4). (F and G) Mouse bone marrow-derived macrophages (BMMs) were treated with GM-CSF (10 ng/mL) and Dex (100 nM) alone or together for 16 h. F) CCL17 mRNA expression and G) secreted CCL17 protein (n = 5). The data are graphed as scatterplots with bars indicating mean ± SEM. ND, not detected. p values were obtained using one-way ANOVA with Tukey post-test, where ∗p < 0.05, ∗∗p < 0.01, ∗∗∗p < 0.001 and ∗∗∗∗p < 0.0001.

Human monocytes were cultured for 16 h in GM-CSF and Dex alone or together, and gene expression was examined by microarray analysis. GM-CSF treatment of monocytes resulted in 164 differentially expressed genes and as previously reported;^6^ *CCL17* was the most highly upregulated gene by GM-CSF (Figure 1A and Table S1). Strikingly, Dex co-treatment antagonized all GM-CSF-regulated genes. Dex-mediated inhibition of *CCL17*, along with other key cytokines, including *TNF*,^6^ was validated by qPCR (Figure 1B). Further, Dex-treated monocytes expressed increased levels of *IL10*, which was downregulated when co-treated with GM-CSF. Unlike TNF and IL10, which were not detected in the GM-CSF-treated monocyte cultures (data not shown), high levels of CCL17 were found in the supernatant (Figure 1C). Consistent with mRNA expression, secreted GM-CSF-induced CCL17 was inhibited when monocytes were co-treated with Dex.

To examine whether the Dex-mediated inhibition of CCL17 expression could be via the glucocorticoid receptor (GR), the steroid receptor antagonist, mifepristone, was utilized. Pre-treatment of monocytes with 1 μM mifepristone for 30 min before stimulation with either GM-CSF alone or together with Dex resulted in abrogation of GC-mediated inhibition of CCL17 expression, at both the mRNA (Figure 1D) and protein (Figure 1E) levels. GM-CSF also dramatically upregulated CCL17 mRNA (Figure 1F) and protein (Figure 1G) in mouse bone marrow-derived macrophages (BMMs), and its expression was again downregulated by Dex.

### Dexamethasone inhibits GM-CSF-induced IRF4 expression via suppressing JMJD3 expression and activity

**Figure 2:**
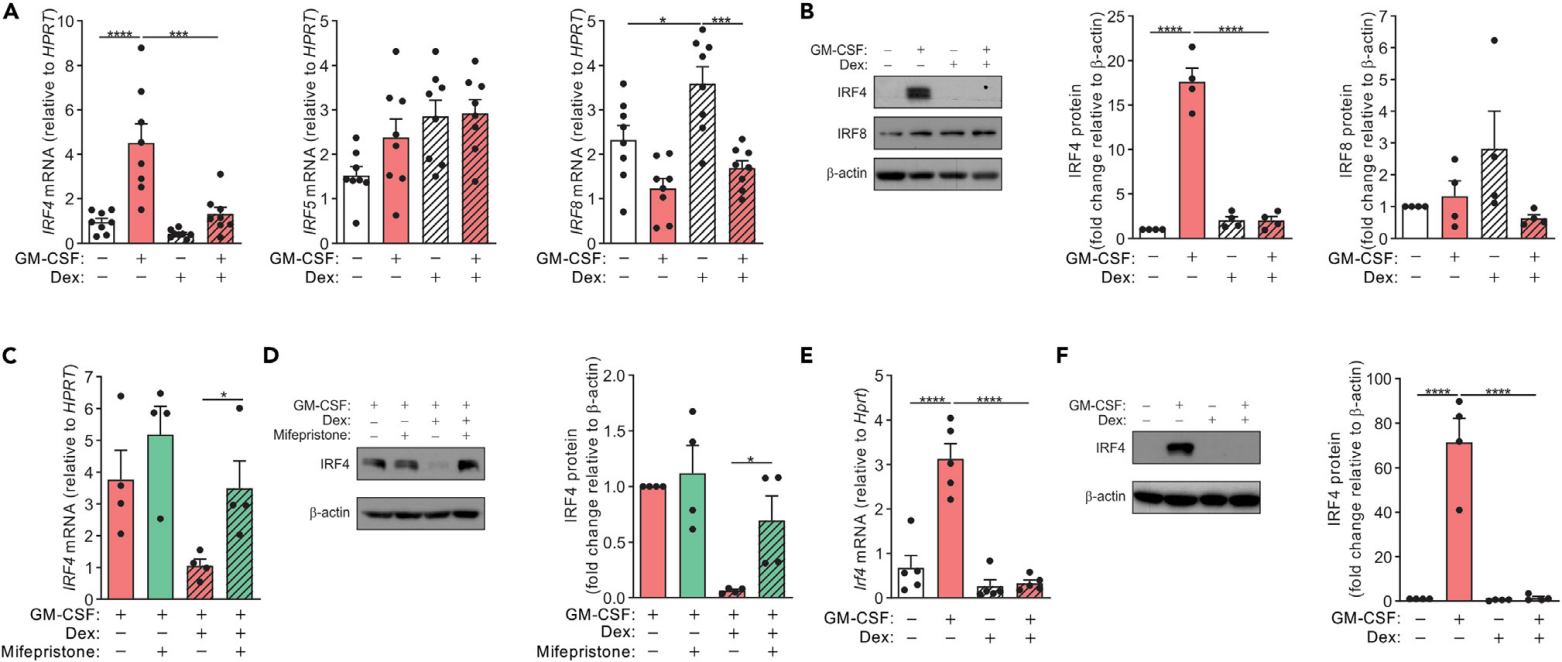
Dexamethasone downregulates GM-CSF-induced IRF4 expression in human monocytes and mouse macrophages (A and B) Human monocytes were treated with either GM-CSF (20 ng/mL) and Dex (100 nM) alone or together for 16 h. A) *IRF4*, *IRF5*, and *IRF8* mRNA expression (n = 8) and B) protein expression in whole cell lysates (western blotting) with anti-IRF4, anti-IRF8, and anti-β-actin antibodies (n = 4). (C and D) Human monocytes were pre-treated with mifepristone (1μM) for 30 min before treated with GM-CSF alone or together with Dex for 16 h. C) *IRF4* mRNA expression (n = 4) and D) IRF4 and β-actin protein expression in whole cell lysates (n = 4). (E and F) BMM were treated with GM-CSF (20 ng/mL) and Dex (100 nM) alone or together for 16 h. E) *Irf4* mRNA expression (n = 5) and F) IRF4 and β-actin protein expression in whole cell lysates (n = 4). The data are graphed as scatterplots with bars indicating mean ± SEM. p values were obtained using one-way ANOVA with Tukey post-test, where ∗p < 0.05, ∗∗∗p < 0.001 and ∗∗∗∗p < 0.0001. Original Western blots are presented in Figure S1.

We next explored the molecular pathway(s) that may be involved in Dex-mediated inhibition of CCL17. We have previously shown that GM-CSF could upregulate CCL17 in an IRF4-dependent manner in both human monocytes and mouse macrophages.^6^ GM-CSF-induced IRF4 expression was inhibited by Dex at the mRNA (Figure 2A) and protein (Figure 2B) levels. IRF5 expression, which was found to have no role in regulating GM-CSF-induced CCL17 expression,^6^ was not affected by the presentence of Dex (Figure 2A). In contrast, IRF8 expression was upregulated by Dex, but GM-CSF downregulated its expression when monocytes were co-treated with Dex (Figures 2A and 2B). Consistent with the CCL17 expression data (Figures 1D and 1E), pre-treatment of monocytes with mifepristone resulted in the Dex-mediated downregulation of IRF4 expression being reversed in monocytes co-treated with GM-CSF (Figures 2C and 2D). Paralleling the human monocyte data, GM-CSF treatment of BMM resulted in a marked increase of IRF4 mRNA and protein, which were dramatically inhibited when the cells were co-treated with Dex (Figures 2E and 2F), again correlating with CCL17 expression (Figures 1F and 1G).

**Figure 3:**
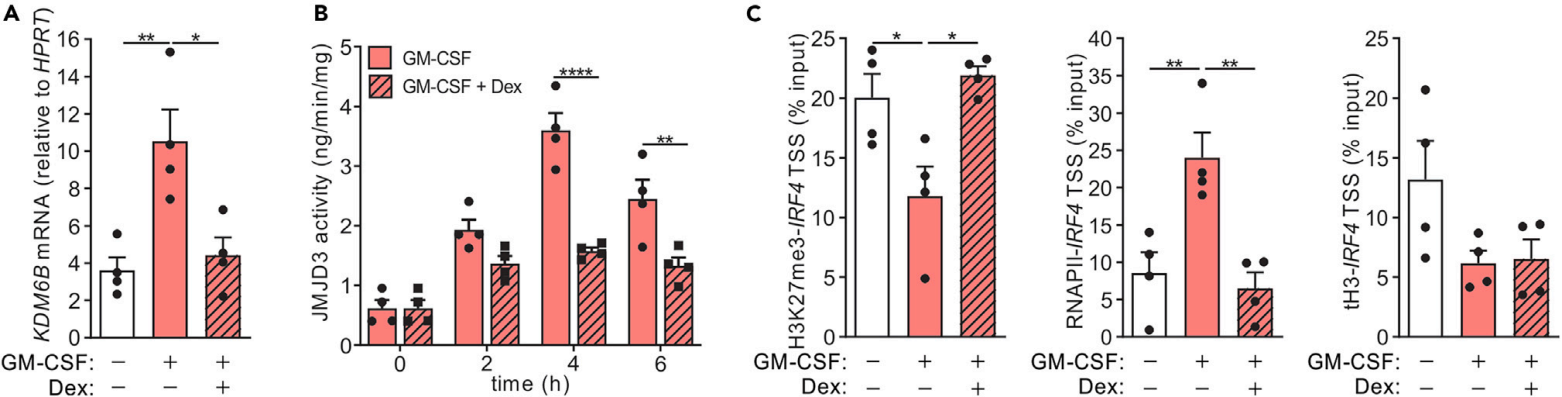
Dexamethasone inhibits JMJD3 expression and activity in human monocytes (A and B) Human monocytes were treated with either GM-CSF (20 ng/mL) alone or together with Dex (100 nM) for 0, 2, 4, and 6 h. A) *KDM6B* (at 4h) mRNA expression (n = 4) and B) JMJD3 activity were determined (n = 4). (C) Human monocytes were treated with GM-CSF alone or together with Dex for 1 h. ChIP analysis of the association of RNA Pol II, H3K27me3, and total H3 with the *IRF4* TSS is expressed as percentage of input DNA (n = 4). The data are graphed as scatterplots with bars indicating mean ± SEM. p values were obtained using either one-way ANOVA with Tukey post-test (A, C) or two-way ANOVA with Sidak post-test (B), where ∗p < 0.05, ∗∗p < 0.01 and ∗∗∗∗p < 0.0001.

In steady state, the promoter region of *IRF4* gene is enriched with the repressive trimethylated histone 3 Lys27 (H3K27me3) mark, which can be catalyzed by the JMJD3 demethylase to its monomethylated state (i.e., H3K27me1), thereby enhancing *IRF4* gene transcription.^10^ We have previously shown that GM-CSF could upregulate JMJD3 expression and activity to control IRF4 expression in human monocytes.^6^ We therefore hypothesized that Dex might regulate JMJD3 expression and/or activity in order to control IRF4 transcription. GM-CSF treatment of monocytes significantly upregulated *KDM6B* mRNA, which was inhibited when monocytes were co-treated together with Dex (Figure 3A). Consistent with its expression data, the GM-CSF-upregulated JMJD3 enzymatic activity was also inhibited by Dex (Figure 3B). Furthermore, chromatin immunoprecipitation (ChIP) assays confirmed that Dex treatment of monocytes blocked the recruitment of RNA polymerase II to the *IRF4* TSS and prevented the GM-CSF-induced loss of H3K27me3 association to this locus, while not altering the total H3 at this site (Figure 3C).

### Dexamethasone ameliorates arthritic pain-like behavior and disease, correlating with decreased CCL17 expression

**Figure 4:**
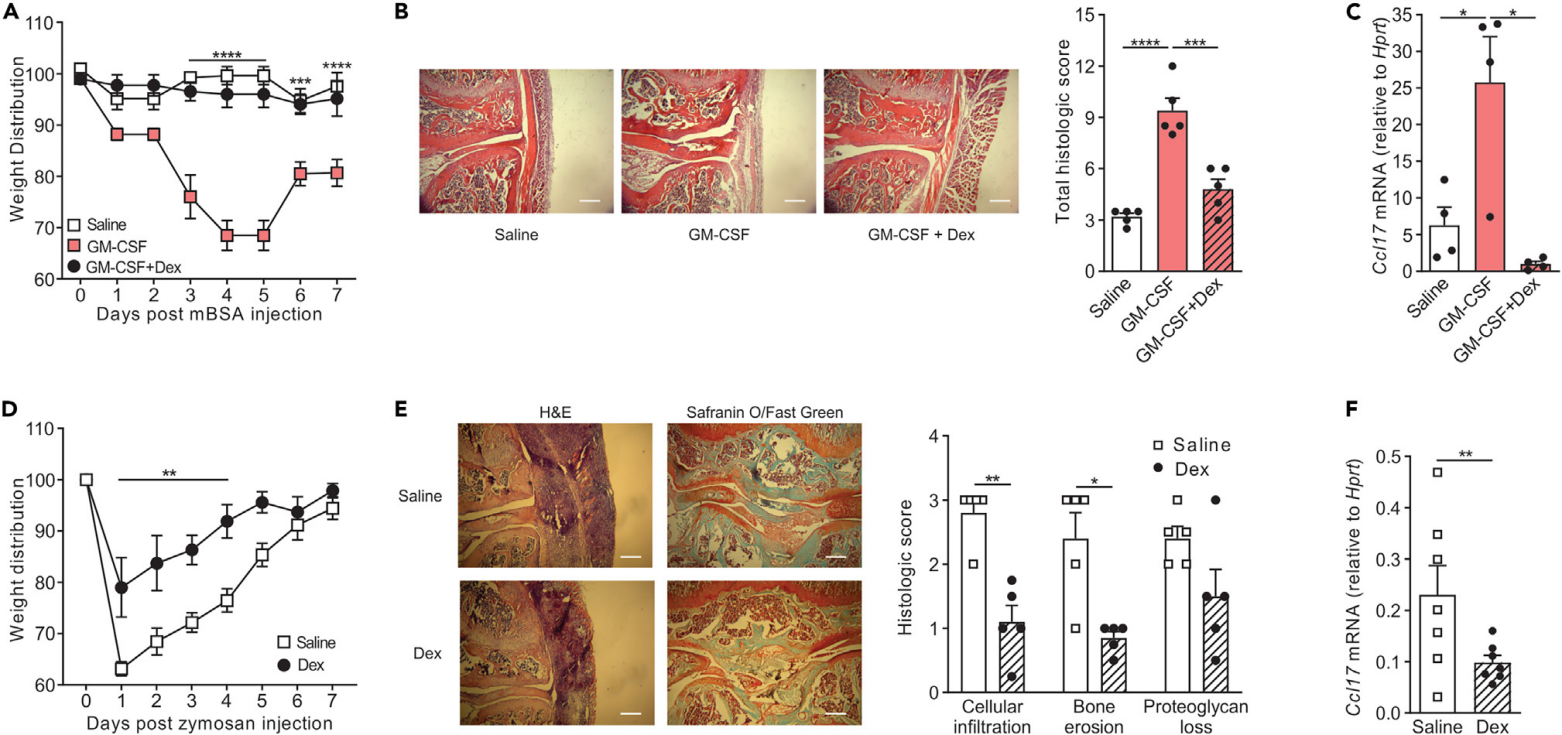
Dexamethasone ameliorates arthritic pain-like behavior and disease, correlating with decreased CCL17 expression in joints (A–C) mBSA/GM-CSF arthritis (mBSA i.a.; day 0) and s.c. saline or GM-CSF (500 ng, days 0–2) or i.p. Dex (0.25 mg/kg, at day −2, 0 and 2) was induced in WT C57BL/6 mice. A) Arthritic pain-like behavior (incapacitance meter; n = 5/group), B) disease (day 7, histology – H&E stain; n = 5/group) and (C) joint *Ccl17* mRNA expression (day 7, qPCR; n = 4/group) were measured. (D–F) Zymosan-induced arthritis (300 μg zymosan i.a.; day 0) or i.p. Dex (0.2 mg/kg, at day −1, 1 and 4) was induced in WT C57BL/6 mice. D) Arthritic pain-like behavior (incapacitance meter; n = 7/group), E) disease (day 7, histology – H&E stain and Safranin O/Fast Green stain; n = 5/group) and (F) joint *Ccl17* mRNA expression (day 7, qPCR; n = 7/group) were measured. The data are graphed with bars indicating mean ± SEM. p values were obtained using two-way ANOVA with either Dunnett post-test (A) or Sidak post-test (D), one-way ANOVA with Tukey post-test (B, C), or unpaired t test (E, F), where ∗p < 0.05, ∗∗p < 0.01, ∗∗∗p < 0.001 and ∗∗∗∗p < 0.0001. Scale bar represents 25 μM.

We have previously shown that GM-CSF-driven and zymosan-induced arthritic pain-like behavior and disease were dependent on CCL17 with both readouts and the latter model being also dependent on GM-CSF.^6^^,^^11^ Given our findings that GM-CSF-induced CCL17 formation can be inhibited by Dex treatment in human monocytes and mouse macrophages, we therefore investigated whether Dex can ameliorate arthritic pain-like behavior and disease utilizing both of these models.^11^^,^^12^^,^^13^ Dex was administered intraperitoneally, and arthritic pain-like behavior was monitored by a change in weight distribution using an incapacitance meter, as before.^6^^,^^7^^,^^8^^,^^9^ GM-CSF-driven arthritic pain-like behavior was ameliorated in mice receiving Dex treatment, and their weight distribution was similar to that of mice that received control saline (Figure 4A). Histological analyses of the joints of these mice indicated that the GM-CSF-driven arthritis was inhibited in mice receiving Dex treatment (Figure 4B). Consistent with the decreased arthritic pain-like behavior and disease, Dex-treated mice displayed significantly lower levels of *Ccl17* mRNA in the joints compared to those in untreated arthritic mice (Figure 4C). Consistent with methylated bovine serum albumin (mBSA)/GM-CSF arthritis, mice undergoing zymosan-induced arthritis and receiving Dex treatment exhibited less pain-like behavior compared to those that were given the control saline (Figure 4D); histological analyses of the joints indicated that the zymosan-induced arthritis was also inhibited following Dex treatment (Figure 4E). Again, Dex-treated mice displayed significantly lower levels of *Ccl17* mRNA in the joints compared to those in untreated arthritic mice (Figure 4F).

### Dexamethasone inhibits CCL17 production in PBMCs from RA patients

**Figure 5:**
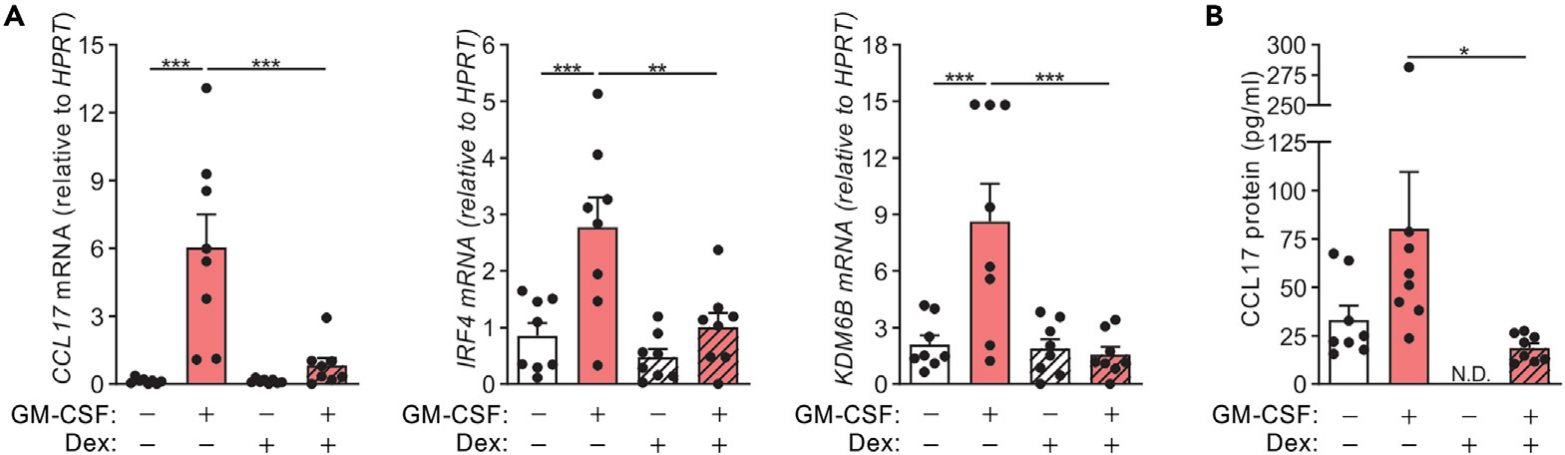
Dexamethasone treatment inhibits CCL17 production in peripheral blood mononuclear cells from RA patients PBMCs from RA patients were treated with either GM-CSF (20 ng/mL) and Dex (100 nM) alone or together for 16 h. (A and B) *CCL17*, *IRF4*, and *KDM6B* mRNA expression and B) secreted CCL17 protein (n = 8) were measured. The data are graphed as scatterplots with bars indicating mean ± SEM. ND, not detected. p values were obtained using one-way ANOVA with Tukey post-test, where ∗p < 0.05, ∗∗p < 0.01 and ∗∗∗p < 0.001.

RA patients are reported to have elevated levels of CCL17 in blood and synovial fluid.^14^ Given that RA patients are often treated with GCs as a first-line therapy, we investigated whether Dex can inhibit CCL17 production in PBMCs from such patients. *CCL17*, *IRF4*, and *KDM6B* mRNA were basally expressed in PBMCs and were further increased following GM-CSF treatment (Figure 5A). On the other hand, Dex treatment inhibited the GM-CSF-induced *CCL17*, *IRF4*, and *KDM6B* mRNA expression. Culturing RA PBMCs resulted in spontaneous secretion of CCL17 protein, which could be inhibited by treating them with Dex alone (Figure 5B). GM-CSF treatment of PBMCs secreted marginally increased levels of CCL17 protein, which was again inhibited when PBMCs were co-treated with Dex (Figure 5B).

## Discussion

GCs are broadly used in the treatment of many autoimmune and allergic diseases due to their potent immunosuppressive and anti-inflammatory functions.^15^ Unfortunately, their long-term usage is often associated with adverse side effects that limit their clinical application. Further examination of the anti-inflammatory functions of GCs is thus warranted. While their diverse modes of actions are still debated, there is a broad consensus that GCs exert their anti-inflammatory actions on monocytes/macrophages by primarily inhibiting the transcription of proinflammatory genes (e.g., *TNF*) possibly by suppressing the NF-κB activity.^16^^,^^17^ Here, we found that GM-CSF-induced CCL17 expression is inhibited by a GC in human monocytes and mouse macrophages. We provide the first evidence that GCs suppress GM-CSF-induced IRF4 expression via downregulating the expression and activity of JMJD3, which demethylates trimethylated-H3K27. Moreover, GC ameliorated GM-CSF-induced and CCL17-dependent inflammatory arthritis and pain development, correlating with a marked decrease in the levels of CCL17 in arthritic joints. Significantly, the CCL17 spontaneously secreted by cultured PBMCs from RA patients was also inhibited by GC treatment.

Gene regulation is finely tuned by a dynamic balance between transcriptionally activating and repressing modifications of histone tails.^18^ It is well established that lysine and arginine methylation can be reversed by evolutionarily conserved enzymes known as histone demethylases.^19^ Trimethylated-H3K27 represents a repressive epigenetic mark, silencing nearby gene expression via the formation of heterochromatic regions. There are only two known H3K27me3 demethylases, JMJD3 and the ubiquitously transcribed tetratricopeptide repeat X chromosome protein (UTX).^20^ In contrast to UTX, JMJD3 is inducible by inflammatory and oncogenic stimuli.^21^^,^^22^ Significantly, it has been proposed that JMJD3, rather than UTX, is involved in the control of inflammation, whereas UTX regulates major developmental processes.^23^^,^^24^^,^^25^ Many studies have also revealed that JMJD3 is a potent enhancer of proinflammatory gene expression, which is typically linked to the activation of NF-κB signaling and subsequent induction of TNF.^21^^,^^26^^,^^27^ While LPS treatment of BMM triggered the recruitment of JMJD3 to the TSSs of many genes, the deletion of JMJD3 impaired the expression of only a limited number of genes, which were mostly inducible inflammatory genes.^21^ GM-CSF has been shown to promote IκB degradation to activate NF-κB signaling pathway,^28^ and we previously demonstrated that LPS treatment of GM-CSF-differentiated mouse bone marrow-derived macrophages (GM-BMMs) showed increased NF-κB activity compared to LPS-treated BMM.^29^ Since Dex can abrogate GM-CSF-induced IκB degradation leading to inhibition of NF-κB signaling,^30^^,^^31^ it could be that Dex suppresses JMJD3 expression via inhibition of GM-CSF-activated NF-κB signaling pathways.

The GR functions as ligand-dependent transcription factor that regulates the expression of GC-responsive genes. Mifepristone is a GR antagonist which prevents GC from binding to its receptor. Our finding that pre-treatment of monocytes with mifepristone restored GM-CSF-induced IRF4 expression and CCL17 production suggests that Dex transcriptionally controls the expression of these key GM-CSF-induced transcriptional and/or epigenetic factors. Indeed, Dex has been shown to suppress JMJD3 gene expression via the binding to both the GRα and the nuclear receptor co-repressor to the negative glucocorticoid response element in the upstream region of the gene.^32^^,^^33^^,^^34^ Our study, indicating that Dex can inhibit GM-CSF-induced JMJD3/IRF4 expression in order to suppress CCL17 production, provides a new mechanism for the anti-inflammatory actions of GCs.

We and others have previously shown that inhibiting JMJD3 function, with GSK-J4, can alleviate pain and block arthritis development in mouse models of arthritis.^6^^,^^9^^,^^35^^,^^36^ Our finding that Dex inhibited GM-CSF-induced JMJD3 expression and activity *in vitro* is consistent with Dex administration ameliorating GM-CSF-induced pain-like behavior and disease, correlating with decreased CCL17 expression, in the monoarticular mBSA/GM-CSF-induced and zymosan-induced inflammatory arthritis mouse models. The former model involves systemic (i.e., subcutaneous) administration of a cytokine (GM-CSF here) to mice given mBSA intra-articularly and can allow downstream pathways dependent on the particular cytokine to be delineated (refs); the widely used zymosan-induced arthritis model involves direct intra-articular injection of the arthritogen. Given the heterogeneity of RA, it is important to use different models with distinct mechanisms involved in the initiation of arthritis. It would be of interest to determine whether Dex can control CCL17 expression in other arthritic models, including systemic models, such as collagen-induced arthritis model, by a similar mechanism in their widespread efficacy in controlling arthritic pain and disease. We previously showed that a neutralizing monoclonal antibody against CCL17 protects mice from developing arthritic pain and disease in various mouse models.^9^^,^^37^ Cyclooxygenase 2 (COX2) is known to be involved in the production of many pain-inducing eicosanoids (e.g., PGE2), which are highly elevated in the synovial tissues and fluid of RA patients.^38^ Furthermore, PGE2 is also elevated in the knee joints of mice with antigen-induced arthritis.^7^ Dex can inhibit COX2 and PGE2 at the transcriptional and post-transcriptional levels, respectively,^39^^,^^40^ and we have previously shown that CCL17-driven inflammatory pain and arthritic pain are indeed dependent on COX2,^6^ suggesting that CCL17 may have a key role in COX2-dependent pain development. Matrix metalloproteinases (MMPs) can play a key role in cartilage destruction, and they are overexpressed in RA.^41^^,^^42^ Notably, GM-CSF can promote the production of several MMPs^43^^,^^44^ and Dex has been shown to inhibit their formation in response to other stimuli.^45^^,^^46^ A role for JMJD3 in regulating MMP2, MMP3, and MMP9 gene expression was demonstrated in endothelial cells,^47^ and, importantly, Dex could downregulate these MMPs via the suppression of JMJD3 expression.^34^ Given the aforementioned results, the prevention of GM-CSF-driven inflammatory arthritis by Dex may partly be mediated by the suppression of MMP production through the downregulation of JMJD3 expression and activity.

RA is characterized by debilitating, painful inflammation which, if left untreated, causes irreversible joint damage. The initial treatment often commences with the use of cDMARDs, such as methotrexate. If the patients are not responsive or unable to tolerate cDMARDs, they may be switched to biologic DMARDs (bDMARDs). Anti-TNF drugs are often the first type of bDMARDs administered to RA patients, but nearly 40% of the patients do not respond or develop resistance over time to anti-TNF therapies.^48^^,^^49^ Significantly, these unresponsive patients were found to benefit more with an alternative bDMARD, rather than a new anti-TNF inhibitor.^50^^,^^51^ Many studies have reported increased levels of GM-CSF and CCL17 in RA patients compared to healthy controls, and therefore targeting these cytokines may be beneficial.^5^^,^^52^^,^^53^ Indeed, anti-GM-CSF therapies have shown promising results in clinical trials.^4^^,^^5^^,^^54^ Mavrilimumab targets the α-subunit of the GM-CSF receptor and has been shown to significantly decrease disease activity in RA patients.^55^ Furthermore, a comparison study showed that mavrilimumab, and not anti-TNF therapeutics, suppressed CCL17 serum levels in RA patients.^55^^,^^56^ This relationship between GM-CSF and CCL17 can be used to measure the efficacy of other anti-GM-CSF therapies since decreased CCL17 serum levels in RA patients correlate with increased therapeutic efficacy.^5^^,^^55^ Therefore, in addition to being a drug target, CCL17 serum levels could serve as a prognostic biomarker enabling clinicians to choose the appropriate bDMARD.^57^

In summary, we have delineated a new anti-inflammatory molecular mechanism for GCs, utilizing human monocytes, mouse macrophages, and an inflammatory arthritis animal model as well as RA patient samples. We have provided evidence for the first time that GCs can inhibit GM-CSF-upregulated epigenetic and transcription factors, JMJD3/IRF4, to suppress the formation of the proinflammatory chemokine, CCL17. Our findings suggest that CCL17 may be a potential therapeutic target in many inflammatory conditions where GCs are used as an anti-inflammatory drug, but without the associated adverse side effects.^3^

### Limitations of the study

While our study provides molecular evidence that GCs can suppress GM-CSF-mediated upregulation of IRF4 and CCL17 expression via downregulating JMJD3 expression and activity, there can be other epigenetic and transcriptional factors that may be controlled by Dex, which can contribute to the suppression of CCL17 formation. Further, we demonstrate a decrease in arthritic pain and disease development in mice receiving Dex administration, correlating with decreased CCL17 expression. Although we have previously demonstrated a non-redundant role for CCL17 in mediating arthritic pain and disease in several mouse models,^6^^,^^8^^,^^9^^,^^37^ there can be several other factors, including other cytokines, which can also be responsible for the disease development that may be suppressed by Dex treatment.

## STAR★Methods

### Key resources table

**Table:** 

REAGENT or RESOURCE	SOURCE	IDENTIFIER
**Antibodies**		

IRF-4 (D9P5H) Rabbit	Cell Signaling Technology	Cat#15106; RRID:AB_2798709
IRF-8 (D20D8) Rabbit	Cell Signaling Technology	Cat#5628; RRID:AB_10828231
β-Actin (AC-74) Mouse	Merck	Cat#A5316; RRID:AB_476743
Rpb1 CTD (4H8) Mouse	Cell Signaling Technology	Ca#2629; RRID:AB_2167468
Trimethyl-Histone H3 (Lys27) Rabbit	Merck	Cat#07-449; RRID:AB_310624
Histone H3 (A3S) Rabbit	Merck	Cat#05-928; RRID:AB_492621

**Chemicals, peptides, and recombinant proteins**		

Recombinant human GM-CSF	R&D Systems	Cat# 215-GM
Dexamethasone	Merck	Cat#D4902
Mifepristone	Merck	Cat#M8046
Zymosan A from Saccharomyces cerevisiae	Merck	Cat#Z4250

**Critical commercial assays**		

EZ-Magna ChIP™ A/G ChIP Kit	Merck Millipore	Cat#17-10086
SensiFAST™ Probe Hi-ROX Kit	Meridian Bioscience	Cat#BIO-82020
Pierce™ BCA Protein Assay Kit	ThermoFisher	Cat#23227
Mouse CCL17/TARC DuoSet ELISA	R&D Systems	Cat#DY529
Human CCL17/TARC DuoSet ELISA	R&D Systems	Cat#DY364
RosetteSep™ Human Monocyte Enrichment Cocktail	StemCell Technologies	Cat#15068
ISOLATE II RNA Mini Kit	Meridian Bioscience	Cat#BIO-52073
Epigenase JMJD3 Demethylase Kit (Colorimetric)	EpigenTek	Cat#P-3084
Chromatin Immunoprecipitation (ChIP) Assay Kit	Merck	Cat#17-295
SensiFAST™ SYBR® Hi-ROX Kit	Meridian Bioscience	Cat#BIO-92020

**Deposited data**		

ArrayExpress	www.ebi.ac.uk/biostudies/arrayexpress/studies/E-MTAB-2212?query=E-MTAB-2212	RRID:SCR_002964

**Experimental models: Organisms/strains**		

C57BL/6J	The Jackson Laboratory	RRID:IMSR_JAX:000664

**Oligonucleotides**		

CCL17_ taqman gene expression probe	ThermoFisher	Assay ID: Hs00171074_m1
TNF_ taqman gene expression probe	ThermoFisher	Assay ID: Hs00174128_m1
IL10_ taqman gene expression probe	ThermoFisher	Assay ID: Hs00961622_m1
IRF4_ taqman gene expression probe	ThermoFisher	Assay ID: Hs00180031_m1
IRF5_ taqman gene expression probe	ThermoFisher	Assay ID: Hs00158114_m1
IRF8_ taqman gene expression probe	ThermoFisher	Assay ID: Hs00175238_m1
KDM6B_ taqman gene expression probe	ThermoFisher	Assay ID: Hs00996325_g1
HPRT_ taqman gene expression probe	ThermoFisher	Assay ID: Hs02800695_m1
IRF4 TSS Forward 5’-ccacctcgcactctcagttt-3′	Sigma-Aldrich	N/A
IRF4 TSS Reverse 5’-ctggaggtcgaacctctggt-3′	Sigma-Aldrich	N/A

**Software and algorithms**		

GraphPad Prism 9.5.1	www.graphpad.com	RRID:SCR_002798
Bioconductor R package	www.bioconductor.org/packages/release/bioc/html/limma.html	RRID:SCR_006442
RUV R package	www-personal.umich.edu/∼johanngb/ruv/index.html	N/A

**Other**		

RPMI-1640 medium	ThermoFisher	Cat#52400041
Fetal Bovine Serum	Merck	Cat#F9423
GlutaMAX™ Supplement	ThermoFisher	Cat#35050061
Penicillin-Streptomycin	ThermoFisher	Cat#15140122
cOmplete™ Protease Inhibitor Cocktail	Roche	Cat#11697498001
NuPAGE™ 10%, Bis-Tris, 1.5 mm, 15-well	ThermoFisher	Cat#NP0303BOX
Pierce™ ECL Western Blotting Substrate	ThermoFisher	Cat#33209
Oligo(dT)	Invitrogen	Cat#18-418-012
SuperScript™ II Reverse Transcriptase	Invitrogen	Cat#18-064-071

### Resource availability

#### Lead contact

Further information and requests for resources and reagents should be directed to and will be fulfilled by the lead contact, A/Prof. Adrian Achuthan (aaa@unimelb.edu.au).

#### Materials availability

This study did not generate new unique reagents.

### Experimental model and study participant details

#### mBSA/GM-CSF-induced arthritis model

mBSA/GM-CSF-induced arthritis model experiments involving mice were approved by the University of Melbourne Animal Ethics Committee (10321) and all experiments conform to the relevant regulatory standards. Monoarticular arthritis was induced in wild-type C57BL/6 female mice as before^6^^,^^13^ by intra articular (i.a.) injection of 100 μg mBSA in 10 μl saline into the right knee on day 0, the left knee being injected with saline, followed by a subcutaneous (s.c.) injection, in the scruff of the neck on days 0-2, of either GM-CSF (500 ng, R&D Systems) or saline. Where applicable, Dex (0.25 mg/kg) was administered on days -2, 0 and 2 by intraperitoneal (i.p.) injection. On day 7, arthritic joints were collected for histologic analysis and gene expression.

#### Zymosan-induced arthritis model

Zymosan-induced arthritis model experiments involving mice were approved by the University of Melbourne Animal Ethics Committee (2022-23414) and all experiments conform to the relevant regulatory standards. For the induction of the zymosan-induced arthritis model,^6^^,^^7^ wild-type C57BL/6 female mice (8-10 weeks) were injected with 300 μg of sonicated zymosan (Sigma-Aldrich) in a 10 μl volume into the left knee joint, while the contralateral knee received saline as a control. Where applicable, Dex (0.2 mg/kg) was administered on days -1, 1 and 4 by i.p. injection. On day 7, arthritic joints were collected for histologic analysis and gene expression.

#### Primary human and mouse cells

Human monocytes were isolated from buffy coats, which were sourced ethically as approved by the University of Melbourne Human Research Ethics Committee (2021-20542), and their research use was in accord with the terms of the informed consents obtained by Australian Red Cross Lifeblood.

Peripheral blood mononuclear cells were isolated from blood samples obtained from RA patients (4 males and 4 females), with disease activity score above 3 (i.e., DAS28-CRP > 3), were taken with informed written consent and their research use was approved by the Royal Melbourne Hospital Human Research Ethics Committee (HREC/14/MH/125). All investigations involving patient samples were conducted according to the Declaration of Helsinki principles.

Experiments involving mouse bone marrow-derived macrophages were approved by the University of Melbourne Animal Ethics Committee (2021-20398) and all experiments conform to the relevant regulatory standards.

### Method details

#### Isolation and culture of human monocytes

Human monocytes were isolated as described before.^58^ Briefly, they were purified from buffy coats, using RosetteSep Ab cocktail (Stem Cell Technologies, Vancouver, BC, Canada), which negatively selects CD14^+^ monocytes, followed by Ficoll-Paque density gradient centrifugation. They were cultured in RPMI 1640, supplemented with 10% heat inactivated fetal bovine serum, 2 mM GlutaMax-1 (Life Technologies, Carlsbad, CA), 100 U/ml penicillin, and 100 mg/ml streptomycin. Isolated monocytes were treated with human GM-CSF (20 ng/ml, R&D Systems, Minneapolis, MN) or dexamethasone (Dex) (100 nM, Sigma-Aldrich, St. Louis, MO), either alone or in combination for indicated time periods. Where applicable, human monocytes were pre-treated with mifepristone (1 μM, Sigma-Aldrich, St. Louis, MO) for 30 min before treated with either GM-CSF alone or together with Dex.

#### Isolation and culture of peripheral blood mononuclear cells from RA patients

Peripheral blood mononuclear cells (PBMCs) from the patient blood were separated using density gradient centrifugation. Briefly, whole blood was diluted with PBS (1:1) and layered on top of Lymphoprep™ medium in a SepMate™ 50ml tube (Stem Cell Technologies) prior to centrifugation. The buffy coat layer containing the enriched PBMCs was collected, and the cells were incubated with warm red blood cell lysis buffer for 10 minutes before being washed with cold PBS. Isolated PBMCs were cultured in RPMI 1640, supplemented with 10% heat inactivated FCS, 2 mM GlutaMax-1 (Life Technologies), 100 U/ml penicillin, and 100 mg/ml streptomycin and were treated with human GM-CSF (20 ng/ml, R&D Systems) or Dex (100 nM, Sigma-Aldrich), either alone or in combination for 16 h.

#### Isolation and culture of bone marrow-derived mouse macrophages

Mouse bone marrow-derived macrophages (BMM) were prepared as previously described.^6^ Briefly, bone marrow cells were isolated from femurs of C57/BL6 wild-type female mice and cultured in RPMI 1640 medium supplemented with 10% heat-inactivated fetal bovine serum, 2 mM GlutaMax-1, 100 U/ml penicillin, and 100 mg/ml streptomycin in the presence of human M-CSF (5,000 U/ml, Chiron, Emeryville, CA). At day 4, nonadherent cells were collected and cultured for a further 3 days again in M-CSF (5,000 U/ml) to derive BMM. The BMM were then stimulated with recombinant mouse GM-CSF (20 ng/ml, R&D Systems) alone, or together with Dex (100 nM), in the absence of M-CSF, for indicated periods of time.

#### Microarray analyses

RNA was isolated from three independent human monocyte preparations, each derived from a single donor and integrity was analyzed using an Experion automated electrophoresis station (Bio-Rad, Hercules, CA). Microarray analyses were preformed following recommended protocols supplied by Agilent Technologies (Santa Clara, CA, USA) as before.^6^^,^^59^ Briefly, Total RNA (1 μg) was used as starting material, which was amplified using the Low RNA Input Linear Amplification Kit (Agilent Technologies). The labeled cDNA was purified using an RNeasy Mini Kit (Qiagen, Hilden, Germany), and eluted and quantified using a NanoDrop ND-1000 spectrophotometer (Thermo Fisher Scientific Inc, Waltham, MA). Following fragmentation, the cRNA was hybridized to a whole human genome microarray (Agilent Technologies) containing 43,376 probes corresponding to 41,264 transcripts. Microarrays were then scanned using a DNA microarray scanner, Model G2565A (Agilent Technologies). Microarray intensity data were read using the read.maimages function from the Limma Bioconductor R package with background correction using the normexp method (https://www.bioconductor.org/packages/release/bioc/html/limma.html). Fold changes, p values and false discovery rates (FDR) were obtained using the RUVinv function from ruv R package^60^^,^^61^ (http://www-personal.umich.edu/∼johanngb/ruv/index.html). The list of control genes was from *Eisenberg* et al*.*^62^ GM-CSF-regulated genes were identified as those with FDR < 0.05 and absolute log fold change > 1. Log fold changes, p values and FDRs of the GM-CSF-regulated protein coding genes and those in Dex alone and Dex-cotreated conditions are provided in Table S1. The data set and technical information compliant with minimum information about a microarray experiment (MIAME)^63^ can be found at the ArrayExpress Archive Web site (https://www.ebi.ac.uk/biostudies/arrayexpress/studies/E-MTAB-2212?query=E-MTAB-2212).

#### Quantitative PCR

Total RNA was extracted using ISOLATE II RNA Mini Kit (Bioline, London, UK) and reverse transcribed using SuperScript III reverse transcriptase (Invitrogen). Quantitative PCR (qPCR) was performed using the QuantStudio 5 Real-Time PCR System (Applied Biosystems, Carlsbad, CA) and predeveloped TaqMan probe/primer combinations for human and mouse *CCL17*, *TNF*, *IL10*, *IRF4*, *IRF5*, *IRF8*, *KDM6B* and *HPRT* (Applied Biosystems). Threshold cycle numbers were transformed to cycle threshold values, and the results were plotted using GraphPad Prism version 9.5.1.

#### ELISA

Secreted human and mouse CCL17 (R&D Systems) were measured by ELISA as per manufacturer’s instructions on a Varioskan Lux Plate Reader (Thermo Fisher).

#### Western blotting

Whole-cell extracts were lysed and Western blotted as described previously.^64^ Briefly, protein concentrations of the samples were determined with a Bio-Rad protein assay kit. Equal amounts of protein were loaded on 10% NuPAGE gels (Invitrogen). The separated proteins were transferred onto a polyvinylidene fluoride membrane and then Western blotted with appropriate antibodies. Antibodies were against IRF4 (D9P5H), IRF8 (D20D8) (Cell Signaling Technologies, Danvers, MA), and β-actin (A5316) (Sigma-Aldrich, St. Louis, MO).

#### JMJD3 activity assay

JMJD3 activity assay was performed as described previously.^64^ Briefly, human monocytes were lysed following treatment with GM-CSF (20 ng/ml) alone or together with Dex (100 nM), and nuclear fractions enriched with NE-PER Nuclear and cytoplasmic extraction reagents (Thermo Fisher Scientific, Waltham, MA). 10 μg of nuclear extracts was subjected to the JMJD3 activity assay with a 120-minute incubation period, using a colorimetric Epigenase JMJD3 Demethylase Activity Assay Kit (Epigentek, Farmingdale, NY). The demethylated product was ascertained from the optical density at 450 nm using a standard curve, and JMJD3 activity (ng/min/mg) calculated as demethylated product (ng) divided by incubation time (min) and input nuclear extract (mg).

#### ChIP assay

ChIP assays were performed as described previously.^64^ Briefly, human monocytes were treated with GM-CSF (20 ng/ml) alone or together with Dex (100 nM) for 1 h before crosslinking protein-DNA complexes with 1% formaldehyde for 10 minutes at room temperature. ChIP was performed with a ChIP Assay Kit (17-295, Millipore) as per the manufacturer’s instructions. Immunoprecipitation was performed with 1μg of anti-RNA Pol II (#2629, Cell Signaling Technologies), anti-H3K27me3 (07-449, Millipore), anti-histone H3 (05-928, Millipore) antibodies, followed by reversal of cross-linking. Immunoprecipitated DNA fragments were then amplified by qPCR with a SensiFAST SYBR Hi-ROX Kit (Bioline) and the following specific primers for human IRF4 transcription start site (TSS) (forward 5′-ccacctcgcactctcagttt-3′ and reverse 5′-ctggaggtcgaacctctggt-3′). Enrichment of histones and RNA Pol II at the gene loci was expressed as percentage of input DNA.

#### Assessment of pain-like behavior and histology

In arthritis mouse models, as an indicator of pain-like behavior, the differential distribution of weight on hind limbs was measured using an incapacitance meter (IITC Life Science Inc, USA).^12^^,^^65^^,^^66^ As an indicator of arthritic pain-like behavior, the differential distribution of weight over a 3 second period between the arthritic limb (i.e., mBSA- or zymosan-injected joint) relative to the non-arthritic limb (i.e., saline-injected joint) was measured. Mice were acclimatized to the incapacitance meter on at least three occasions prior to the commencement of the experiment. Three measurements were taken for each time point and averaged.

On day 7, mice were sacrificed, and knee joints collected, fixed, decalcified, and paraffin embedded.^12^^,^^66^ Frontal sections (7 μm) were stained with H&E or Safranin O/Fast Green. For the mBSA/GM-CSF model, cellular infiltration, synovitis (synovial hyperplasia), pannus formation, cartilage damage and bone erosions were scored separately from 0 (normal) to 5 (severe).^12^ For the zymosan-induced arthritis model, cellular infiltration, bone erosions and proteoglycan loss were scored separately from 0 (normal) to 3 (severe) as before.^6^^,^^7^

### Quantification and statistical analysis

Statistical analyses were performed using two-way ANOVA with Dunnett/Sidak post-test for comparing two different groups over a constant period of time, one-way ANOVA with Tukey post-test for more than two different groups or unpaired t-test for comparing mean of two independent groups, as indicated. A P value < 0.05 indicates significance. Data were graphed as scatter plots with bars indicating mean ± SEM from at least four independent experiments using GraphPad Prism version 9.5.1.

## Data Availability

• Microarray data have been deposited at ArrayExpress and are publicly available as of the date of publication. Accession numbers are listed in the key resources table. • This paper does not report original code. • Any additional information required to reanalyze the data reported in this paper is available from the lead contact upon request.

## Supplemental information

Document S1. Figure S1

Table S1. The effects of Dex on GM-CSF-regulated genes in human monocytes, related to Figure 1A
