# Supplementary material for: Epigenetic and transcriptional regulation of CCL17 production by glucocorticoids in arthritis
Source: iScience. 2023 Sep 27;26(10):108079. doi: 10.1016/j.isci.2023.108079 (PMC10583050; doi:10.1016/j.isci.2023.108079)
Supplement: Document S1. Figure S1 [file mmc1.pdf]

iScience, Volume *26*

Supplemental information

Epigenetic and transcriptional regulation of CCL17 production by glucocorticoids in arthritis

Tanya J. Lupancu, Kevin M.C. Lee, Mahtab Eivazitork, Cecil Hor, Andrew J. Fleetwood, Andrew D. Cook, Moshe Olshansky, Stephen J. Turner, Richard de Steiger, Keith Lim, John A. Hamilton, and Adrian A. Achuthan

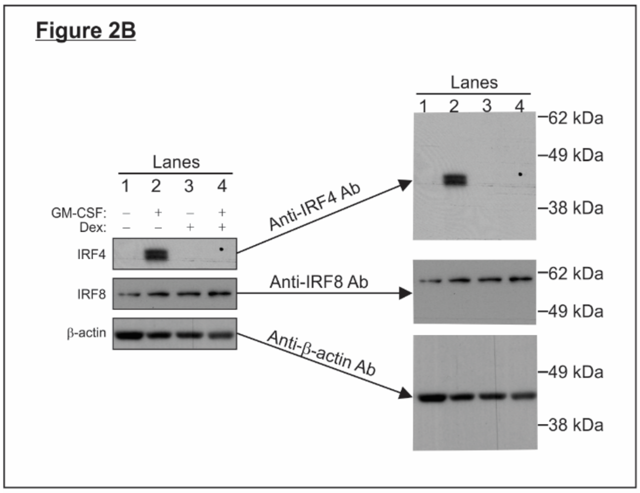

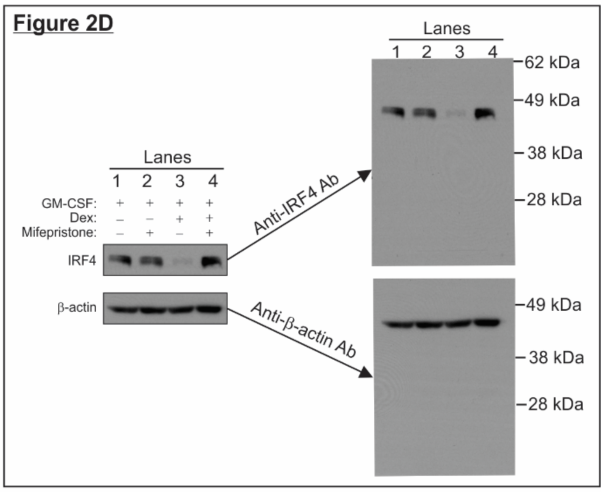

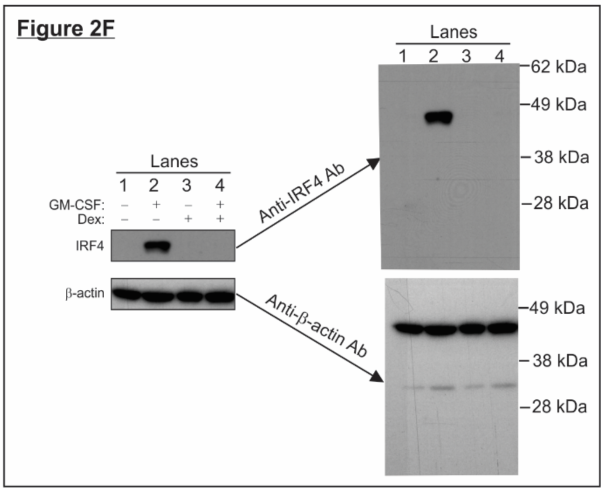

Figure S1: Original Western blot scans, related to Figure 2B, D and E.
